# One year comprehensive prospective follow-up of achalasia patients after peroral endoscopic myotomy

**DOI:** 10.1080/07853890.2021.2005253

**Published:** 2021-11-21

**Authors:** Helge Evensen, Milada Cvancarova Småstuen, Anselm Schulz, Vendel Kristensen, Lene Larssen, Jorunn Skattum, Olav Sandstad, Truls Hauge, Asle W. Medhus

**Affiliations:** a Department of Gastroenterology, Oslo University Hospital, Oslo, Norway; b Faculty of Medicine, University of Oslo, Oslo, Norway; c Faculty of Health Sciences, Oslo Metropolitan University, Oslo, Norway; d Department of Radiology and Nuclear Medicine, Oslo University Hospital, Oslo, Norway; e Department of Diagnostic Physics, Norwegian Imaging Technology Research and Innovation Center (ImTECH), Oslo University Hospital, Oslo, Norway; f Unger-Vetlesen Institute, Lovisenberg Diaconal Hospital, Oslo, Norway; g Department of Abdominal and Pediatric Surgery, Oslo University Hospital, Oslo, Norway

**Keywords:** Achalasia, peroral endoscopic myotomy, objective outcome evaluation, predictive outcome factors, gastroesophageal reflux disease

## Abstract

**Background and aims:**

Peroral endoscopic myotomy (POEM) is an established therapy for achalasia, but outcome evaluation has often been limited to Eckardt score (ES). The present study was aimed to improve knowledge about outcome evaluation and predictive outcome factors by performing a comprehensive objective evaluation of achalasia patients treated by POEM.

**Methods:**

This single centre prospective study reports outcome data 12 months after POEM in treatment-naive achalasia patients. A predefined follow-up protocol included ES, high resolution manometry, 24-h pH measurement, upper endoscopy and timed barium esophagogram (TBE). Univariate and multivariate regression analyses were performed to analyze association between post-POEM variables and identify predictive factors for objective outcome.

**Results:**

Fifty patients were included with a drop-out rate of <5%. ES, lower oesophageal sphincter pressures, TBE heights and maximal oesophageal diameter were all reduced after POEM (*p* < .001), and 28% (13/47) of the patients had a positive 24-h pH registration. An oesophageal diameter >3 cm after POEM was associated with treatment failure assessed by ES (*p* = .04) and TBE (*p* = .03). Advanced achalasia stage (*p* = .02) and long symptom duration (*p* = .04) were identified as independent predictive factors for poor outcome assessed by TBE.

**Conclusions:**

The present study confirms that POEM is an efficient therapy for achalasia. The comprehensive objective evaluation after POEM demonstrates that long symptom duration and major changes in oesophageal anatomy at diagnosis imply poor treatment outcome, and a post-POEM dilated oesophagus is associated with treatment failure.

**Key messages**
- Peroral endoscopic myotomy (POEM) is established as a safe and effective therapy for achalasia.
- Timed barium esophagogram offers objective variables that are valuable in treatment response evaluation.
- Advanced achalasia stage and long symptom duration are predictive factors for poor objective treatment response after POEM.

## Introduction

Achalasia is a rare, primary oesophageal motility disorder, characterized by impaired oesophageal body peristalsis and relaxation of the lower oesophageal sphincter (LES) [1]. The aim of achalasia treatment is to reduce LES pressure, thereby facilitating bolus transport and alleviating symptoms.

Pneumatic balloon dilatation (PD) and laparoscopic Heller myotomy (LHM) are established and effective achalasia treatment modalities [2]. During the last decade, peroral endoscopic myotomy (POEM) has become one of the first-line therapies for achalasia [3].

Symptoms of achalasia including dysphagia, regurgitation and retrosternal pain, are together with weight loss reported as the Eckardt score (ES) [4]. The score was developed as a standardized symptom registration tool for achalasia patients treated with PD, and further validation data are limited [5,6]. In addition to the mainly symptomatic evaluation of treatment by ES, objective evaluation is warranted, but often limited [7]. Results are frequently reported retrospectively with a short minimum follow-up period and a variable follow up-rate [8]. Furthermore, previous treatment may influence outcome negatively, and it is thus of interest to estimate the outcome of POEM in patients that are treatment-naive [9,10]. Post-POEM reflux should also be addressed, since reports indicate significant reflux after POEM, albeit with a varying prevalence [11].

High-resolution manometry (HRM) and timed barium esophagogram (TBE) provide objective evaluation and are supplements to the ES [12]. There is limited correlation between post-treatment symptoms and TBE, but TBE still predicts the need for re-intervention better than symptoms and HRM [13–15]. Nevertheless, the evaluation of treatment effect after achalasia therapy is, according to clinical experience, difficult and there is a call for an evaluation beyond the mainly symptom-based ES [12].

Therefore, the primary aim of this prospective, single-centre study was to report 12 months subjective and objective outcome variables after POEM in treatment-naive achalasia patients by applying a predefined and standardized comprehensive follow-up protocol.

Our secondary aims were to analyze associations between POEM outcome variables and to identify potential predictive factors for objective outcome after POEM.

## Materials and methods

### Study design and ethics

The present study is a single centre prospective study on consecutive treatment-naive achalasia patients undergoing POEM with 12 months follow-up. Data from standard clinical follow-up were prospectively included in the study database, which was approved for use in research by the institutional review board at Oslo University Hospital (personvern@oslo-universitetssykehus.no and case number 2016/5437). All patients signed informed consent regarding their willingness to include their data in the study database. The study adheres to the Declaration of Helsinki.

### Patients

Achalasia patients diagnosed at Oslo University Hospital were evaluated for eligibility, and the inclusion period was from March 2016 to November 2018. The diagnosis of achalasia was based on HRM findings according to the Chicago classification [16], supplemented by TBE [17] and upper endoscopy (EGD). After confirming the diagnosis, treatment decision was made in a multidisciplinary team meeting (MDT) involving gastroenterologists, upper GI surgeons and radiologists. The preferences of the patients were also included in the treatment decision. When the MDT-meeting recommended a myotomy, most patients were offered POEM due to a low treatment capacity of LHM at the hospital in the study period. LHM was offered to the patients with a clear preference for this procedure. PD was chosen in patients with significant comorbidity.

### Inclusion criteria

Inclusion criteria: Confirmed achalasia, ES >3, treatment-naive, age ≥18 years, decision of POEM in MDT-meeting, American Society of Anesthesiologists (ASA) score ≤3 [18] and written informed consent.

### Follow-up

A predefined post-POEM follow-up protocol was applied with telephone consultations after one week and three and six months. After 12 months, a comprehensive evaluation including HRM, TBE [17], EGD and 24-h ambulatory pH registration (24-h pH) was performed, except in cases of treatment failure where earlier evaluation was required.

### Protocol for POEM

At our department, POEM was an established treatment prior to the inclusion period [19]. A team of experienced endoscopists (HE, LL and TH) with competence in advanced gastrointestinal endoscopy including endoscopic retrograde cholangiopancreatography, stent-treatment and endoscopic submucosal dissection, performed POEM. The POEM procedure was performed as described by Inoue et al. [3]. A submucosal bleb and a mucosal incision was performed 10–12 cm oral to the GE-junction before creating the submucosal tunnel. By default, anterior myotomy was performed proximally from 2 cm distal to the incisional opening to at least 2 cm distal to LES, by visual assessment. Selective myotomy of the circular layer was executed proximally, while all visible muscle fibres were cut at LES level and distally. All lesions suggestive of deeper mucosal injury were registered and treated as perforations.

A regimen of nil per os was applied after POEM until negative barium swallow at postoperative day one. Postoperatively, peroral ciprofloxacin was prescribed twice daily for 5 days, and patients were instructed to adhere to a liquid diet the first 3–5 days. At hospital discharge, a proton pump inhibitor, typically pantoprazole 40 mg daily, was recommended used daily until discontinuation at least one week prior to the 12-months control.

### Symptom registration, objective tests and post-POEM reflux evaluation

ES was registered grading each variable from 0 to 3, with 12 as highest score, indicating severe symptoms at every meal and weight loss of more than 10 kg. ES pre-POEM and after 12 months was registered by the physicians performing HRM (OS, VK and AWM.). Telephone registration of ES at three and six months was performed by one dedicated physician (HE).

TBE was performed with radiographs taken at 1, 2 and 5 min after ingestion of 150 ml barium sulphate suspension, as described by Neyaz et al. [17]. Patients that only tolerated a reduced volume pre-POEM were given a similar volume at post-POEM control. In case of no remaining barium column after 1 min, the diameter was measured based on the remaining barium delineation of the oesophageal contours.

The ManoScan™ ESO High Resolution Manometry System (Medtronic, Minneapolis, MN) was applied. HRM was performed and analyzed according to the Chicago classification, v 3.0, and achalasia was classified in subtypes I, II and III [16].

24-h pH registration was performed using Digitrapper™ pH testing System (Medtronic, Minneapolis, MN). Antiacid medication was withdrawn at least 1 week prior to recordings.

Endoscopy included pictures of the Z-line for reflux evaluation. The grading of reflux esophagitis was based on review of the pictures of the Z-lines by two experienced endoscopists (HE and AWM). At 12-months follow-up, reflux symptoms were recorded.

### Definitions and variables

Definitions and variables applied in the present evaluation are presented in Table 1. Outcome variables applied were: ES, TBE barium height, oesophageal diameter, reduction rate on TBE (RR), LES respiratory mean pressure (LES P), LES 4 s integrated relaxation pressure (LES IRP), distal oesophageal acid exposure time (AET), reflux esophagitis, reflux symptoms, peri- and postprocedural complications.

**Table 1.** Definitions and variables.

Favourable/poor objective response	Timed barium esophagogram reduction rate ≥ 0.5/< 0.5 [20]
Clinical success	Eckardt score ≤ 3 after POEM [2, 5, 7, 21]
Positive 24-h pH	Distal oesophageal acid exposure time > 6 % [22]
Positive upper endoscopy	Grade ≥ A esophagitits [23]
Oesophageal diameter	Maximal diameter on timed barium esophagogram
Reflux symptoms	Heartburn or acid indigestion during the week before 12 months control (yes/no)
Advanced achalasia stage	Achalasia stage > 1 [24]
Long symptom duration	Symptom duration ≥ 5 years
Treatment-naive	No previous surgical or endoscopic achalasia therapy
POEM procedure time (min)	Initial scope insertion to final scope withdrawal
Adverse events	Clavien Dindo classification [25]
Hospital stay (days)	Day before POEM to day of discharge

RR at 1, 2 and 5 min was calculated as follows:

$$1-\frac{\mathrm{postoperative}\;\mathrm{TBE}\;\mathrm{barium}\;\mathrm{height}}{\mathrm{preoperative}\;\mathrm{TBE}\;\mathrm{barium}\;\mathrm{height}}$$

A postoperative barium height > preoperative barium height was classified as RR < 0.5. If the preoperative barium height was 0 cm, RR could not be calculated.

Pre-treatment achalasia stages 1–4 were recorded based on maximum oesophageal diameter and presence of sigmoidization on TBE. The non-sigmoid stages were categorized based on increasing diameter; ≤3 cm (stage 1), >3 to 6 cm (stage 2) and 6–8 cm (stage 3). Stage 4 was defined as a diameter ≥8 cm or any size with sigmoidization [24]. When performing analyses, stage 1 achalasia was categorized as “non-advanced achalasia” and stage >1 as “advanced achalasia.”

### Statistical analysis

Data were described with median and interquartile range (IQR) (continuous variables) and counts with percentages (categorical variables). Crude associations between pairs of categorical data were assessed with Chi-square test or Fisher’s exact test, when appropriate. Continuous data were analyzed using non-parametric methods, Mann–Whitney Wilcoxon test and Wilcoxon signed ranks test. Crude associations between POEM outcome variables were analyzed using univariate binary logistic regression models.

To identify possible predictive factors for poor objective response, we fitted univariate and multiple logistic regression models. In step 1, we performed univariate logistic regression analyses and identified possible predictors that reached *p*-values <.2. Step 2: possible predictors from step 1 were included in a multiple regression model. The results are expressed as odds ratios (OR) with 95% confidence intervals (CI). Further, the results from the final multiple logistic regression model were transformed into probabilities using the following formula:

$$P(Y=1X)=\frac{e^{b_{0+}b_{1+}b_{2+}b_{3+}b_{4+}b_{5+}b_{6}}}{1+e^{b_{0+}b_{1+}b_{2+}b_{3+}b_{4+}b_{5+}b_{6}}\;}$$

The resulting probabilities with 95% CI were arranged in a prediction matrix to provide a visual representation. Given the limited sample size, the lower and upper bounds of CI were constructed using bootstrapping with 10,000 repetitions. All tests were two-sided. *p*-values <.05 were considered statistically significant. The study was considered exploratory and hence no correction for multiple testing was done. All analyses were performed using SPSS ver 25 (SPSS, Chicago, IL).

## Results

**Figure 1.**
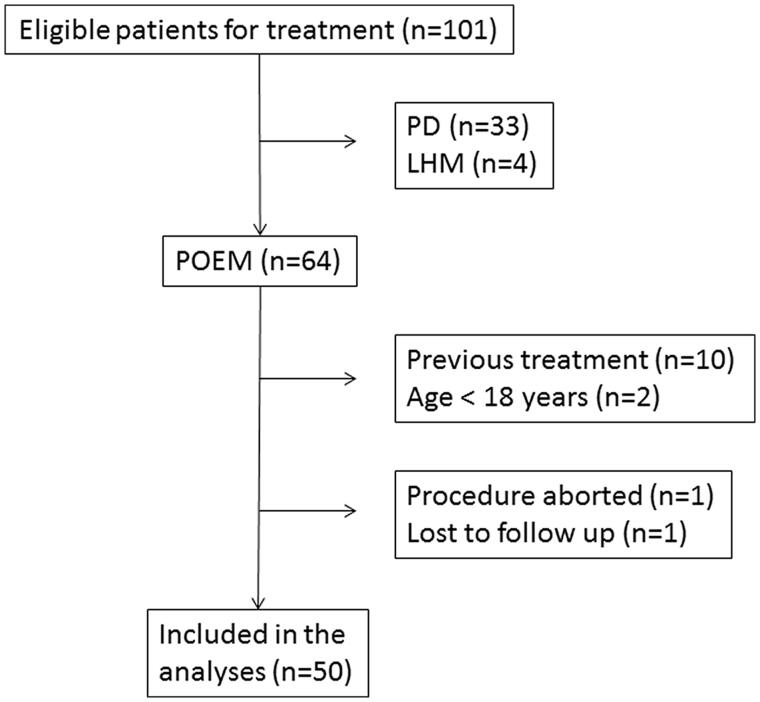
Flowchart of patient recruitment.

In total, 50 treatment naive achalasia patients were included (Figure 1). Baseline data are presented in Table 2. Symptom duration for patients with achalasia stage 1 was 5.0 years (2.0–8.0), and was similar (*p* = .29) to symptom duration for stage > 1 patients, which was 3.0 years (2.0–6.1). Three patients had been treated pharmacologically with calcium channel blocker or nitroglycerine prior to POEM.

**Table 2.** Baseline characteristics (*n* = 50).

	Median (IQR)
Age (years)	47.0 (24.0–70.0)
BMI (kg/m^2^)	23.0 (17.0–30.0)
Symptom duration (years)	3.8 (2.0–7.3)
	*n* (%)
Female gender	26 (52)
ASA score:	
1	16 (32)
2	31 (62)
3	3 (6)
Achalasia subtype:	
I	7 (14)
II	39 (78)
III	4 (8)
Achalasia stage^a^:	
1	23 (47)
2	17 (35)
3	0 (0)
4	9 (18)
Maximal diameter > 3 cm^a^	26 (53)
Subtype I	7 (100)^b^
Subtype II	19 (50)^b^
Subtype III	0 (0)^b^
Sigmoid configuration^a^	9 (18)
Subtype I	6 (86)^b^
Subtype II	3 (8)^b^
Subtype III	0 (0)^b^

^a^*n* = 49.

^b^% of respective subtype.

POEM was performed 78 (30–137) days after the initial TBE. The scheduled 12 months evaluation was performed 357 (350–368) days after POEM (*n* = 48). In two patients, an earlier evaluation with ES, HRM and TBE was required due to clinical failure (89 and 155 days after POEM, respectively), and these data are included in the 12 months analyses.

ES, LES-pressures, TBE barium column heights and maximal oesophageal diameter were all significantly reduced after POEM (Table 3). Clinically successful treatment (ES ≤ 3) was observed in 92% (46/50) of the patients 3 months after POEM and in 86% (43/50) at 12 months. In total, 75% (36/48) of the patients had a favourable objective response (1 min RR ≥0.5 at 12 months).

**Table 3.** Outcome 12 months after POEM.

	Baseline	*n*	12 months	*n*	*p*-value
Eckardt score	8.0 (6.8–9.3)	50	2.0 (0.0–3.0)	50	<.001
Eckardt score > 3					
Stage 1	100%	23	22%	23	<.001
Stage > 1	100%	26	8%	26	<.001
Manometry (mm Hg)					
LES P	50.8 (38.4–62.7)	48	20.8 (14.0–27.0)	49	<.001
LES IRP	33.9 (26.0–38.8)	48	11.0 (7.3–15.6)	49	<.001
Timed barium esophagogram					
1 min (cm)	9.9 (6.9–13.0)	49	2.4 (0.0–4.8)	50	<.001
5 min (cm)	7.0 (3.6–12.4)	49	0.0 (0.0–3.3)	50	<.001
Max diameter (cm)	3.2 (2.5–4.2)	49	2.6 (2.0–3.2)	49	<.001
Reduction rate ≥ 0.5					
1 min			75%	48	
5 min			76%	45	
Reduction rate 1 min					
Stage 1			1.0 (0.6–1.0)	22	= 0.04*
Stage > 1			0.7 (0.2–1.0)	26	
Reduction rate 5 min					
Stage 1			1.0 (0.9–1.0)	18	= 0.25*
Stage > 1			1.0 (0.2–1.0)	26	

Values are median (IQR) unless otherwise stated. LES P: lower oesophageal sphincter respiratory mean pressure; LES IRP: lower oesophageal sphincter 4 s integrated relaxation pressure.

*Stage 1 versus stage >1.

At 12 months, patients with achalasia stage 1 and patients with stage > 1 had similar ES (*p* = .23), whereas 1 min RR was higher in stage 1 patients (*p* = .04). For sigmoid achalasia (stage 4), 78% (7/9) of the patients had ES ≤3 at 12-months control, similar to non-sigmoid patients (*p* = .60).

Twelve months ES was similar across achalasia subtypes (*p* = .20). In subtype I, the frequency of oesophageal dilatation and sigmoidization, respectively, was significantly higher than in subtype II (*p* = .002 and *p* < .001) and in subtype III (*p* = .003 and *p* = .002). Achalasia stages and subtypes were significantly associated (*p* < .001).

Post-POEM oesophageal diameter was significantly associated with both RR and ES, and post-POEM ES was associated with RR (Table 4). Post-POEM LES IRP and AET were not associated with RR or post-POEM ES. There was no association between post-POEM LES IRP and AET (*p* = .25).

**Table 4.** Associations between POEM outcome variables at 12 months. Univariate logistic regression analyses.

	1 min RR		5 min RR		Eckardt score	
	OR [95 % CI]	*p-v*alue	OR [95 % CI]	*p-v*alue	OR [95 % CI]	*p-v*alue
LES IRP	2.12 [0.54; 8.33]	0.28	2.62 [0.58; 11.90]	0.21	6.00 [0.66; 54.24]	0.11
AET	1.65 [0.39; 6.99]	0.50	1.91 [0.35; 10.56]	0.46	1.88 [0.28; 12.78]	0.52
Diameter	9.71 [2.18; 43.48]	0.03	14.93 [2.92; 76.92]	0.01	6.60 [1.05; 41.51]	0.04
Eckardt score	8.47 [1.32; 55.56]	0.02	5.99 [0.85; 41.67]	0.07	( )	

RR: reduction rate on timed barium esophagogram; LES IRP: 4s lower oesophageal sphincter integrated relaxation pressure; AET: distal oesophageal acid exposure time. Cut-offs: RR ≥0.5, ES >3, LES IRP ≥10 mmHg, AET >6 %, diameter >3 cm.

**Figure 2.**
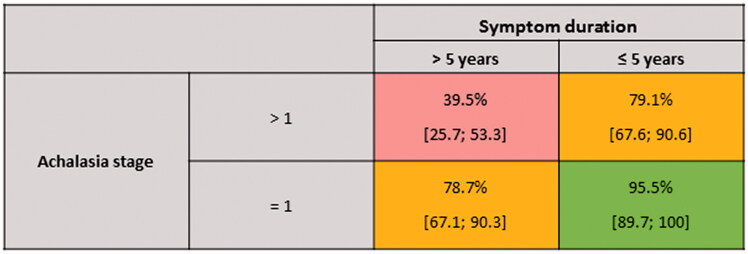
Prediction matrix for favourable objective response (1 min RR ≥ 0.5). Probabilities for each combination of stage and symptom duration are presented with confidence intervals (CI).

When adjusted for height, achalasia stage >1 and symptom duration ≥5 years both remained independent significant predictors of poor objective response (Table 5). Patients with achalasia stage >1 were more than 10 times more likely to experience poor objective response (OR = 10.6; 95% CI [1.40–83.33]) and patients with symptom duration ≥5 years were almost seven times more likely to report poor objective response (OR= 6.67; 95% CI [1.08–41.67]). The results from the multiple regression model were arranged in a prediction matrix. Patients with short symptom duration and stage 1 had 95.5% probability of a favourable objective response (1 min RR ≥0.5), while the probability of a favourable outcome was 39.5% for patients with long symptom duration and stage >1 (Figure 2).

**Table 5.** Risk factors for poor objective response (1 min RR < 0.5).

Variables	OR	95 % CI	*p*-value
*Univariate logistic regression analyses*			
Gender			
Female	2.50	[0.64; 9.82]	.19
Male	1.00		
Age	1.03	[0.98; 1.08]	.27
BMI	1.00	[0.88; 1.14]	.96
Height	1.13	[1.02; 1.25]	.02
Operation time	1.00	[0.99; 1.01]	.66
Symptom duration			
<5 years	1.00		.07
≥5 years	3.53	[0.89; 14.06]	
Achalasia stage			
Stage 1	1.00		.11
Stage >1	3.35	[0.78; 14.46]	
Subtype			
I	5.92	[1.07; 32.26]	.04
II	1.00		
III	2.21	[0.18; 27.78]	.54
*Multivariate logistic regression analyses*			
Achalasia stage			
Stage 1	1.00		.02
Stage >1	10.64	[1.39; 83.33]	
Symptom duration			
<5 years	1.00		
≥5 years	6.67	[1.08; 41.67]	.04
Height	1.16	[1.03; 1.30]	.01

RR: reduction rate on timed barium esophagogram.

Post-POEM reflux data, procedural data and complications related to the POEM procedure are presented in Table 6. Of the patients with positive 24-h pH based on total AET, 54% (7/13) had a positive test in the upright position whereas 77% (10/13) had a positive test in the supine position. Four patients with grade B and two patients with grade C esophagitis had a negative 24-h pH (AET <4%). EGD (positive/negative) and reflux symptoms (yes/no) were significantly associated (*p* = .01), while there was no significant association between 24-h pH (positive/negative) and EGD (*p* = .09) findings or between 24-h pH and reflux symptoms (*p* = .25).

**Table 6.** Procedural data and complications (*n* = 50).

Procedural duration (min)^a^	153 (130–185)
Myotomy length (cm)^a^	11 (11–13)
Hospital stay (days)^a^	2 (2–3)
	*n* (%)
Periprocedural complications	
Mucosal perforation	9 (18)
Incomplete closure of incision	1 (2)
Subcutaneous emphysema	7 (14)
Postoperative complications	
Mediastinitis	1 (2)
Submucosal leak	4 (8)
Post-POEM bleeding	2 (4)
Aspiration pneumonia	1 (2)
Post-POEM reflux	
Upper endoscopy^b^	
LA grade A	10 (20)
LA grade B	10 (20)
LA grade C	4 (8)
24-h pH^c^	
AET >6 %	13 (28)
Reflux symptoms^d^	15 (37)

AET: distal oesophageal acid exposure time; LA: Los Angeles Classification.

^a^Median (IQR).

^b^*n* = 49.

^c^*n* = 47.

^d^*n* = 41.

One serious postoperative complication (Clavien Dindo 3 b) occurred, with prolonged hospitalization due to incomplete incisional closure and development of mediastinitis, which was treated conservatively. Nine suspected or verified mucosal perforations were treated endoscopically with clips. All these patients had post-POEM barium swallow without mediastinal leakage, and received standard post-POEM treatment according to our protocol. The 12 months outcomes were similar in patients with and without periprocedural complications, assessed by ES and TBE.

## Discussion

The present study demonstrates an excellent clinical effect of POEM in treatment naive patients with achalasia and an acceptable rate of adverse events. Post-POEM reflux is prevalent, but generally moderate. The patients’ excellent adherence to the applied follow-up protocol provides a unique and almost complete set of subjective and objective data, revealing an association between post-treatment symptoms (ES), oesophageal diameter and clearance. Moreover, long symptom duration and anatomical alteration of the oesophagus at time of diagnosis imply a poor post-treatment prognosis.

The demonstrated symptomatic effect after POEM is similar to previous results, with an initial clinical success exceeding 90% 3 months after treatment with a slight decrease after 12 months of follow-up [21]. LES pressures and TBE barium heights were also significantly reduced after POEM (Table 3). Moreover, an oesophageal diameter exceeding 3 cm at the 12-months control was associated with treatment failure, assessed by ES and RR (Table 4). This is in line with previous results where a persistent diameter > 3 cm post-LHM was associated with increased risk of re-intervention, and reduction in the barium height and width both indicated therapeutic response due to improved oesophageal clearance [26].

The excellent symptomatic response after POEM may limit statistical comparisons of patients as there are few patients in the group with an ES > 3 after treatment. Furthermore, the definition of clinical success based on a post-treatment ES ≤ 3 seems arbitrary [5]. Questioning the validity of post-treatment evaluation by symptom reporting, studies have demonstrated a discrepancy between symptomatic and objective outcomes. TBE has been identified as the preferred tool for evaluating treatment response and predicting need for reintervention [13,14,20,26]. In line with other studies, 1 min analysis was a sensitive variable for detecting insufficient treatment outcome [14,26]. Hence, we applied 1 min RR as outcome variable to evaluate predictive factors for objective response (Table 5).

Achalasia stage and symptom duration were identified as predictive factors for objective response after POEM. The impact of these predictive factors was quantified and presented visually in a prediction matrix to provide a novel and clinically useful tool for prediction of outcome (Figure 2). It should be noted that the prediction matrix is based on exploratory analyses and a limited number of patients. However, it provides clinically relevant information and should be further explored in larger studies. Most POEM studies include patients with different treatment status, and previous treatment has been demonstrated to influence treatment outcome [10,27]. The present study design was chosen to assess outcome exclusively in treatment-naive patients, and may have been important for identifying predictive factors such as symptom duration, which may be masked by a history of previous treatment. Kumagai et al. applied RR as variable for POEM outcome similarly to our study, identifying younger age as a negative predictive factor, but neither symptom duration nor achalasia stage were included as variables in the analyses [20]. In line with our findings, long symptom duration was identified as a negative predictive factor for symptomatic failure after POEM by Liu et al. [10], and sigmoid achalasia a risk factor for symptomatic failure after LHM in the large study by Zaninotto et al. [28]. Furthermore, Urakami et al. recently demonstrated that advanced achalasia stage is a risk factor for symptomatic failure after POEM and need for reintervention [27].

Further subanalyses with comparison of all four achalasia stages and the three HRM subtypes would have been interesting. However, as achalasia subtypes were significantly associated with achalasia stage, these two variables could not be included in the same statistical model due to multicollinearity. Based on our own experience and previous data on POEM and the different subtypes, we chose to include achalasia stage in the regression analyses as stage was considered more clinically relevant. In contrast to reports after LHM where achalasia subtype III is associated with inadequate treatment response, the limited number of subtype III patients in our study had similar response as the other subtypes [29]. This finding corresponds with several POEM reports, demonstrating excellent and similar symptomatic outcome across all subtypes [30]. The applied achalasia stage categorization was intended to group the patients into two well-defined categories: (i) patients with marked anatomical changes according to degree of oesophageal dilatation and tortuosity and (ii) patients with non-dilated oesophagus and presumably straight configuration (Tables 1, 3 and 5). This grouping of patients with oesophageal dilatation and sigmoidization into one category of special interest for the predictive outcome factors analyses, corresponds well with the results of the study by Urakami et al. [27].

While ES outcome was similar regardless of stage of achalasia, the improvement in oesophageal clearance (RR) was less pronounced in patients with advanced achalasia. This may imply a poorer long term prognosis and a higher risk for future reintervention. The discrepancy between symptoms and objective evaluation in the patients with altered oesophageal anatomy provides new information on POEM in advanced achalasia. Data demonstrating successful POEM treatment of advanced achalasia have been based on improvement of ES [31]. Although one recent study on sigmoid achalasia applied TBE in the outcome evaluation, the methodological difference does not allow comparison with the present study [32].

In our study, AET and LES IRP 12 months after POEM were not associated, nor were they associated with other outcome variables, including ES and reflux symptoms (Table 4). Ponds et al. recently demonstrated that reflux symptoms and oesophageal acidification are often not reflux-related in treated achalasia patients [33]. These findings question the role of pH measurement and HRM in routine follow-up of achalasia patients. Correspondingly, in recent guidelines for routine follow-up, HRM and pH measurement are not recommended [34]. The availability, simplicity and objectivity of TBE suggest that this modality should be the primary follow-up examination combined with ES and EGD to assess treatment response, complications related to the treatment and progression of the underlying condition.

Similar to other studies, our data (Table 6) suggest that post-POEM reflux is prevalent [11] . Our cut-off for positive 24-h pH is in accordance with the recent and rather strict Lyon consensus [22], which might explain the higher endoscopic and symptomatic reflux rates recorded. There was no significant association between findings of 24-h pH and EGD, and of notice, there were several negative 24-h pH recordings in patients with reflux esophagitis. The presence of reflux esophagitis is, however, relevant for long-term complications such as peptic strictures and dysplasia, underlining the role of EGD in follow-up. The demonstrated frequency of post-POEM reflux esophagitis suggests that long-term, regular antiacid medication is indicated in many patients. Moreover, the high prevalence of supine reflux suggests that patients, in whom reflux is suspected, should be recommended to sleep with their torso elevated after POEM.

In the 50 patients included, one serious postoperative complication occurred, whereas minor complications were more prevalent. Periprocedural complications did not affect outcome after 12 months. Although the liberal definition of mucosal perforation may have contributed to the rather high number of minor complications, the present study demonstrates that POEM, similar to LHM and PD, is an invasive therapy with a subsequent risk for adverse events [2]. Furthermore, in our study, anterior myotomy was performed by default, which has been associated with a higher frequency of adverse events including mucosal perforations, than posterior myotomy [35].

In the present study, the relatively small number of study participants represents a study limitation. However, the extraordinary adherence to the study protocol may to a certain degree compensate for this, and follow-up of 50 patients should be sufficient to reveal substantial and clinically relevant findings. Nevertheless, the follow-up does not provide data beyond 12 months and comprehensive long term data are also needed. Post-POEM reflux was evaluated with EGD, 24-h pH and reflux symptoms after a limited period of PPI discontinuation, and a dedicated reflux questionnaire was not applied.

The strengths of the present study are the unique follow-up rate, which is close to 100%, in combination with a comprehensive evaluation protocol both providing baseline and 12 months follow-up data.

The present study confirms that POEM is an efficient therapy for achalasia. After POEM, reflux is prevalent, but usually moderate. Clinically relevant, a dilated oesophagus is associated with treatment failure. Furthermore, long symptom duration and major changes in oesophageal anatomy at diagnosis indicate a poor treatment outcome, suggesting that achalasia patients are preferably treated early in the course of their disease. Although POEM is effective across the different achalasia phenotypes, follow-up and evaluation including TBE seem particularly important in long-standing and advanced achalasia with an increased risk of therapeutic failure.

## Data Availability

Data associated with this study are stored at a local server at Oslo University Hospital and are available from the corresponding author upon reasonable request.
